# Supplementary figures and images for: Purification and biochemical characterization of recombinant Persicaria minor β-sesquiphellandrene synthase
Source: PeerJ. 2017 Feb 28;5:e2961. doi: 10.7717/peerj.2961 (PMC5333544; doi:10.7717/peerj.2961)

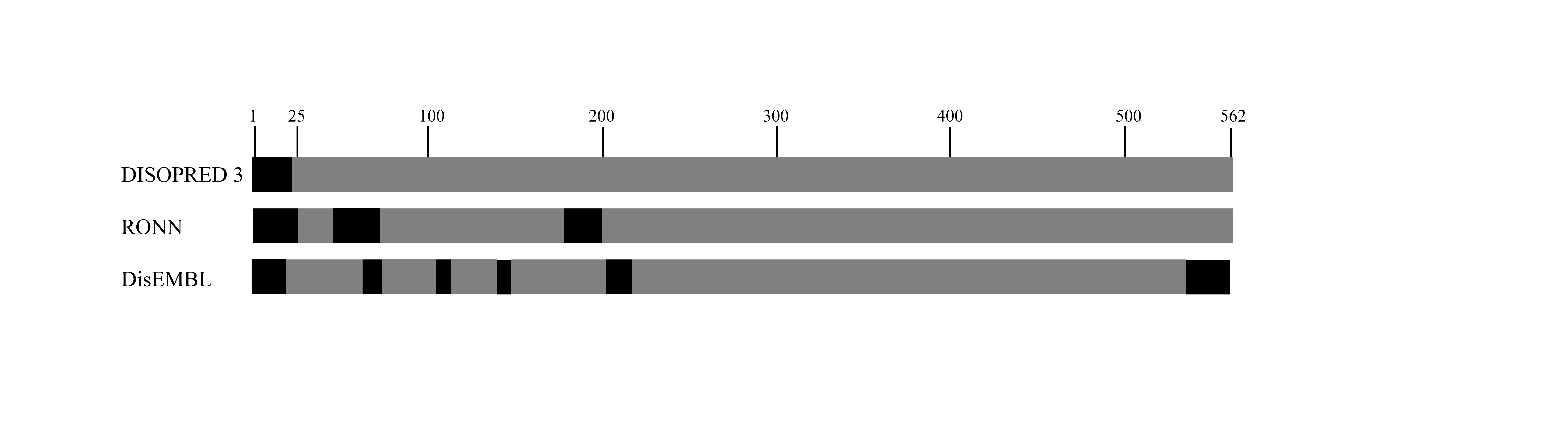

Supplement: Figure S1 — Black- residues predicted to be disordered. Each row represents the results from the disorder prediction server of DISOPRED (Jones & Cozzetto, 2015), RONN (Yang et al., 2005) and DisEMBL (Linding et al., 2003), respectively. [file peerj-05-2961-s001.png]

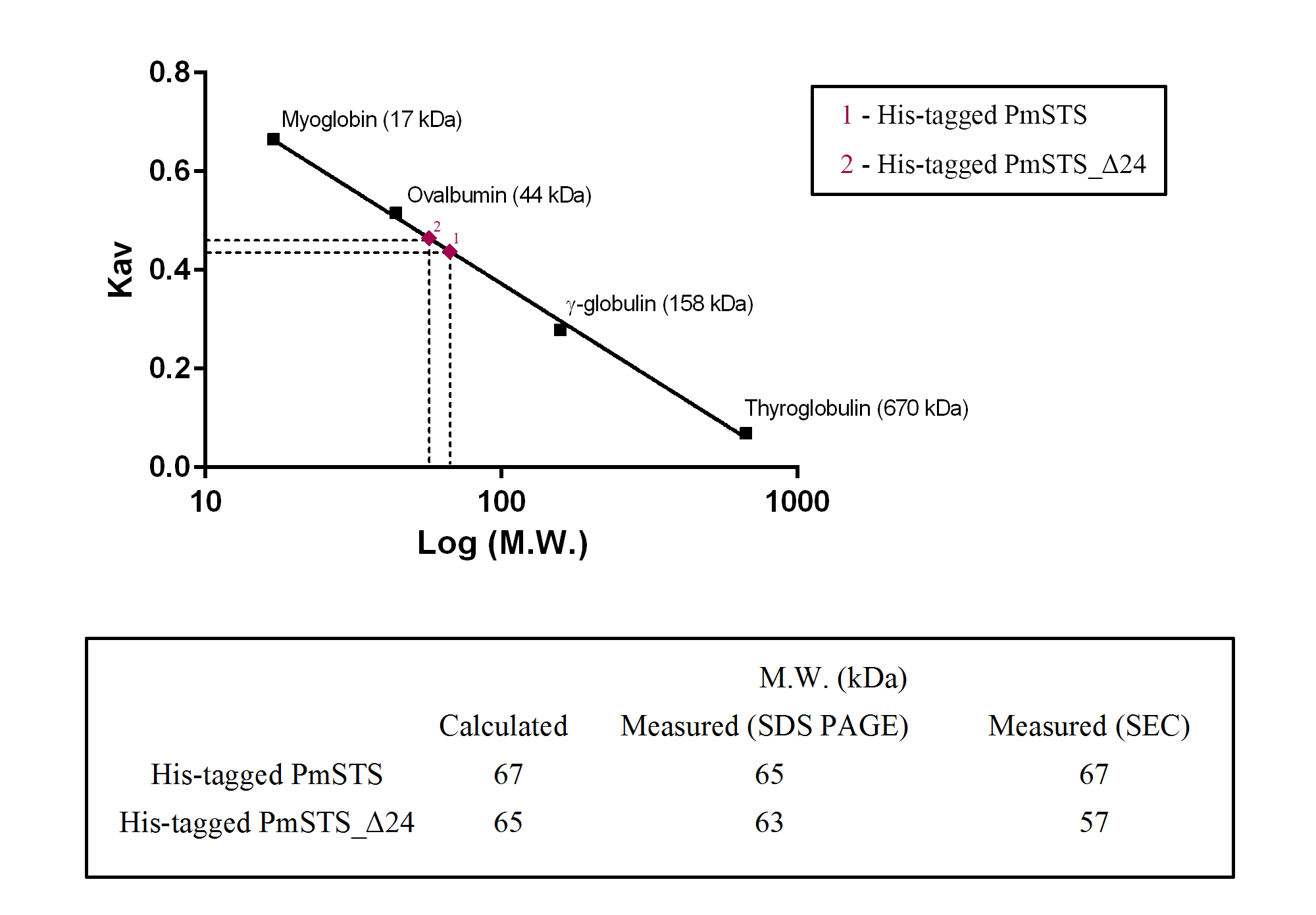

Supplement: Figure S2 — The size exclusion chromatography column was calibrated with the following protein standards: thyroglobulin (670 kDa), γ -globulin (158 kDa), ovalbumin (44 kDa), and myoglobin (17 kDa). The elution pattern of the protein size markers was linear on a semilog plot. Elution data are represented as log molecular weight to Kav. Kav was calculated as in the equation (Ve − Vo)/(Vt − V0), Ve, Elution volume; Vo, Void volume (determined by the elution of Blue dextran, 2,000 kDa); Vt, total column volume. Calculated and measured molecular weight values of His-tagged PmSTS and His-tagged PmSTS_Δ24. [file peerj-05-2961-s002.png]

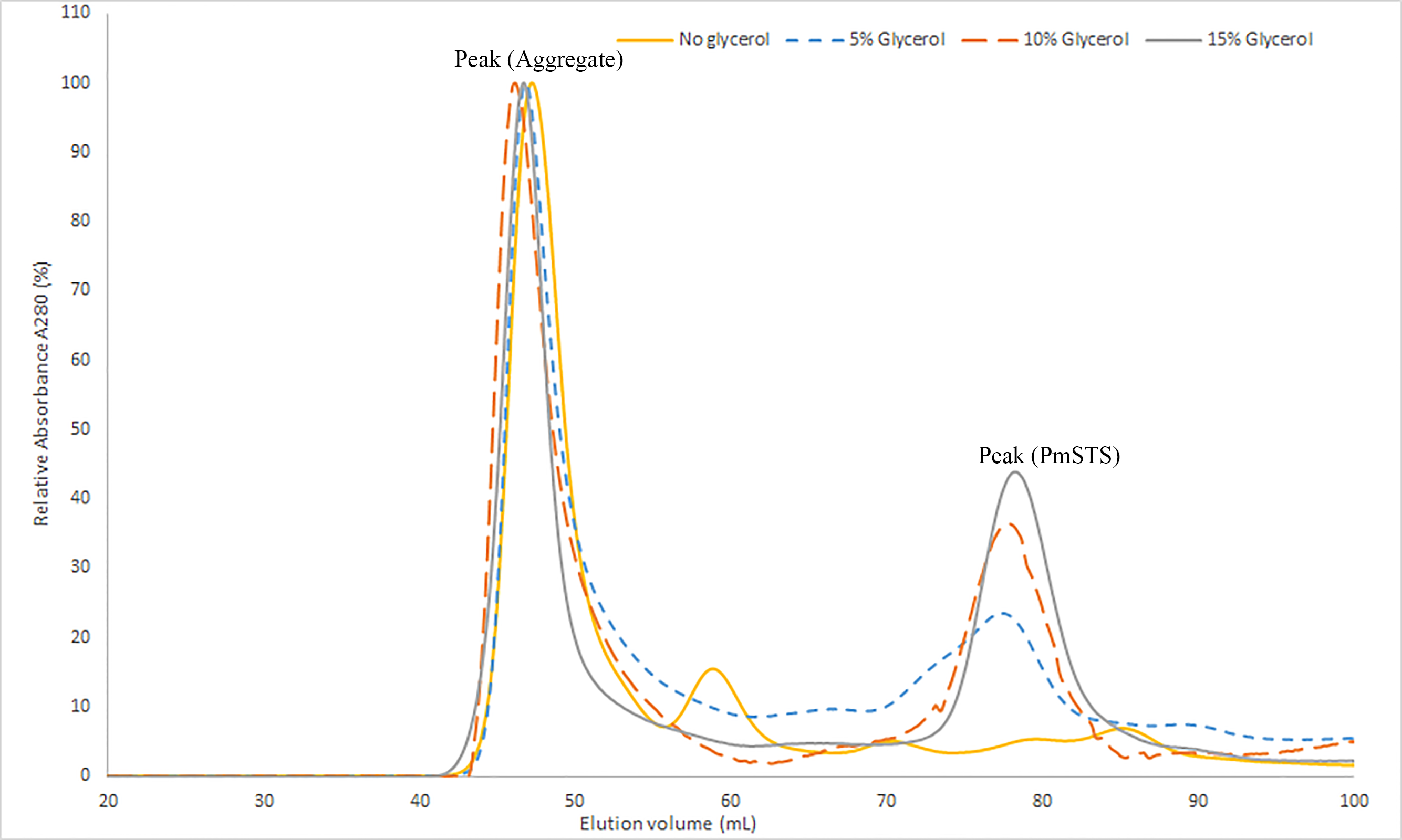

Supplement: Figure S3 — For comparison purpose, all SEC chromatogram is normalized based on the height of peak (Aggregate). [file peerj-05-2961-s003.png]

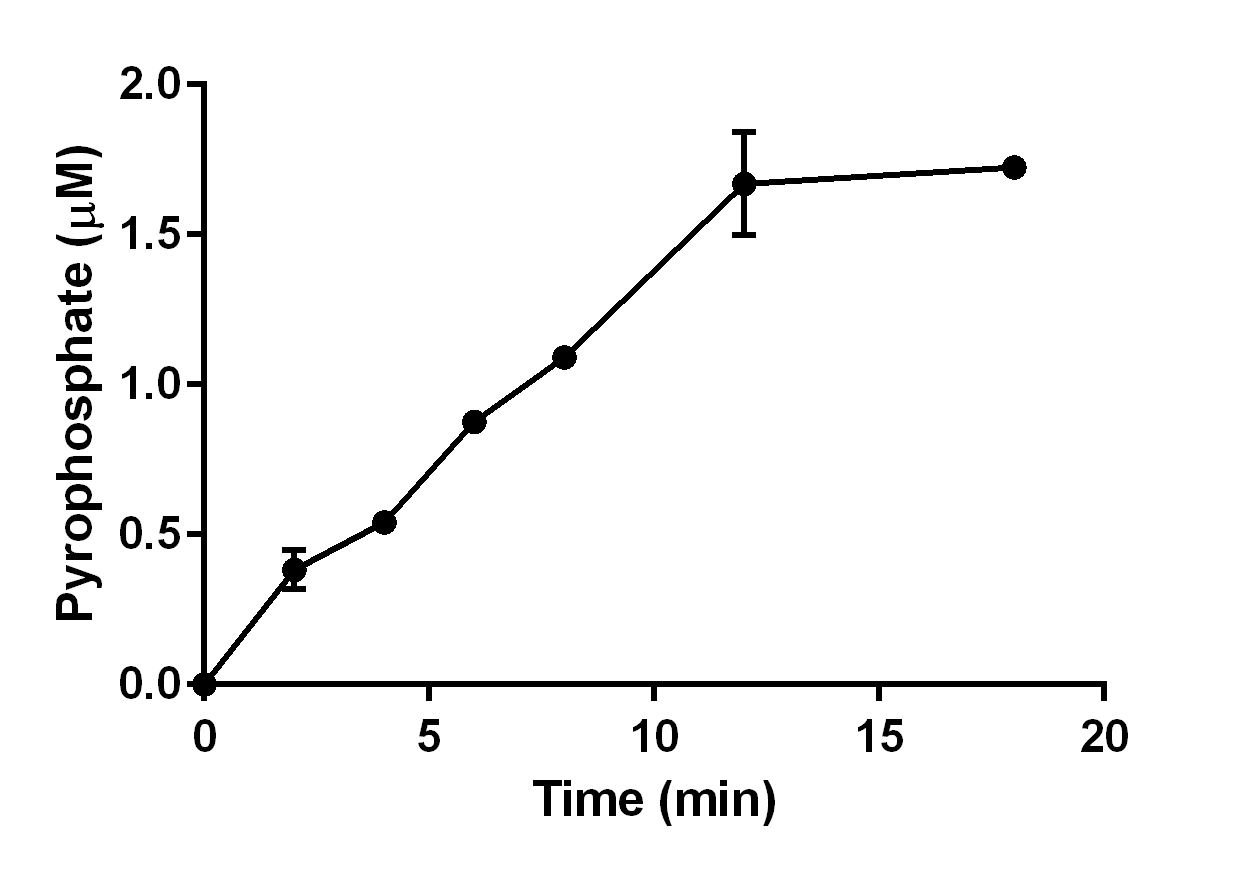

Supplement: Figure S4 [file peerj-05-2961-s004.png]

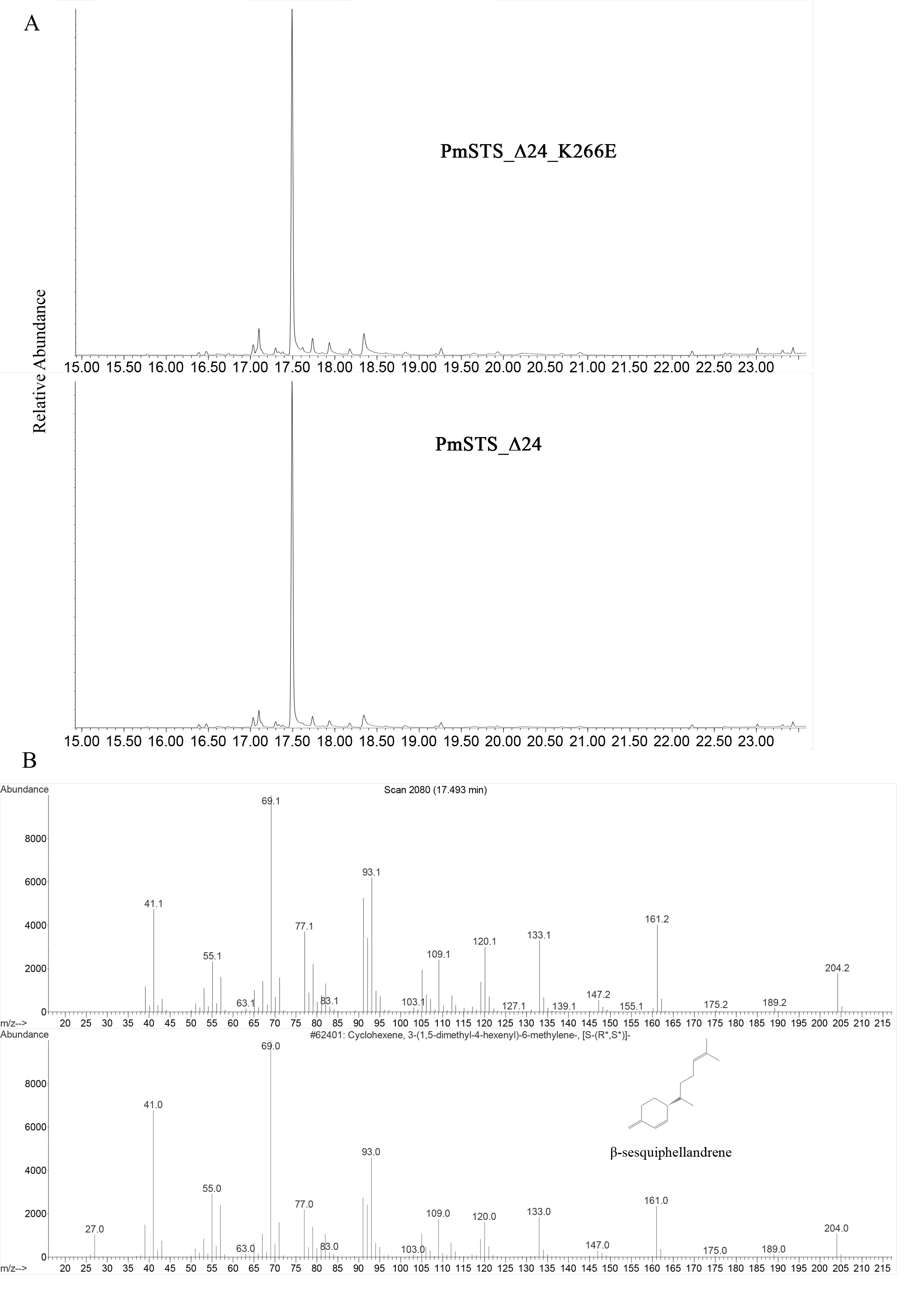

Supplement: Figure S5 [file peerj-05-2961-s005.png]

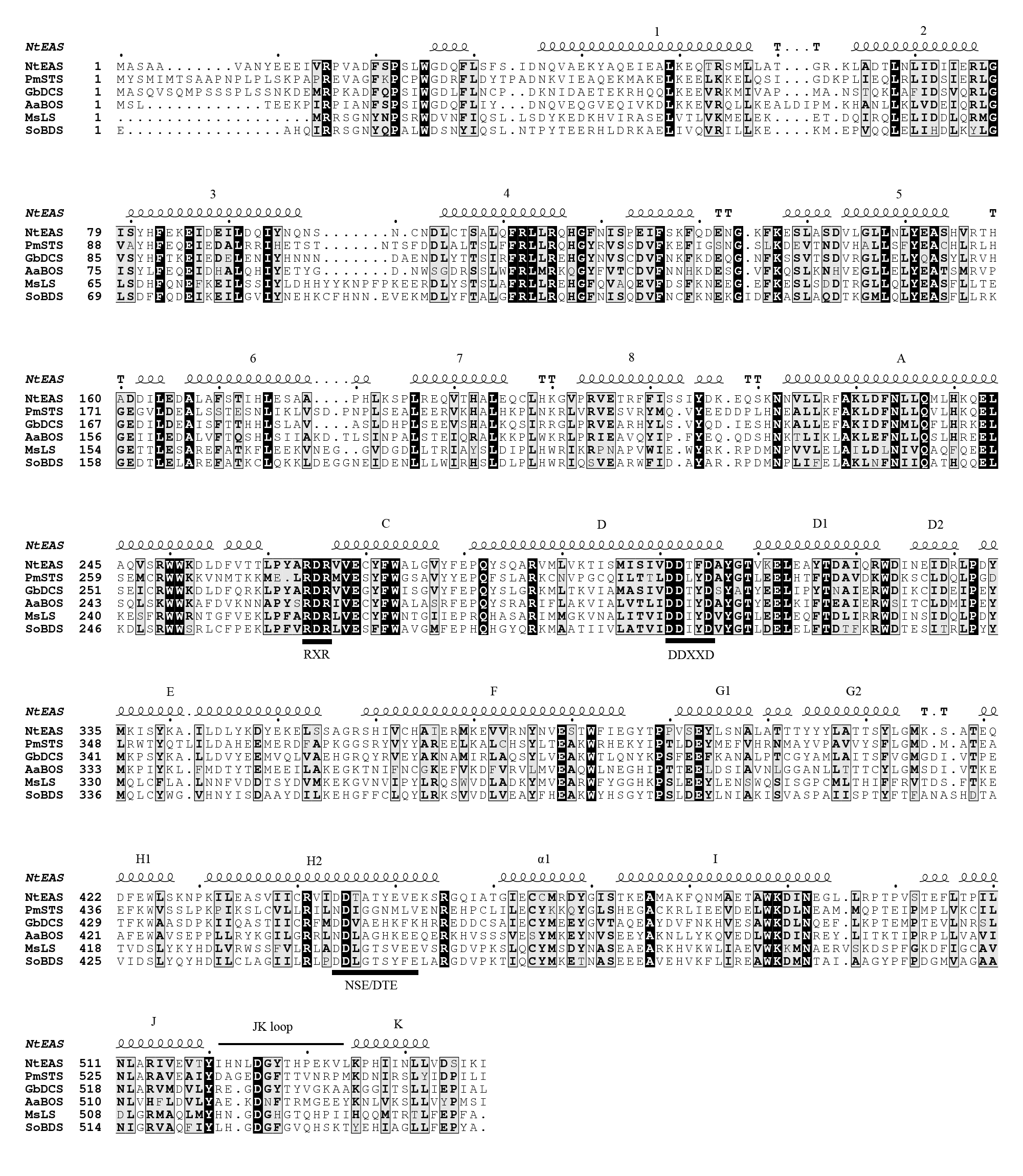

Supplement: Figure S6 — The alignment was generated by T-Coffee and drawn with ESPript. Consensus amino acid residue are boxed in black, secondary structure elements of NtEAS (PDB: 5EAS) are shown above the sequences. The conserved motifs (RXR, DDXXD, and NSE/DTE) are underlined in black. Naming of helices was based on the convention used for NtEAS (Starks et al., 1997). The sesquiterpene synthase sequences aligned are NtEAS (N. tabacum 5-epi-aristolochene synthase), GaDCS (G. arboreum δ -cadinene synthase), AaBOS (A. annua α-bisabolol synthase), MaLS (M. spicata limonene synthase) and SoBDS (S. ofiicinalis bornyl diphosphate synthase). [file peerj-05-2961-s006.png]

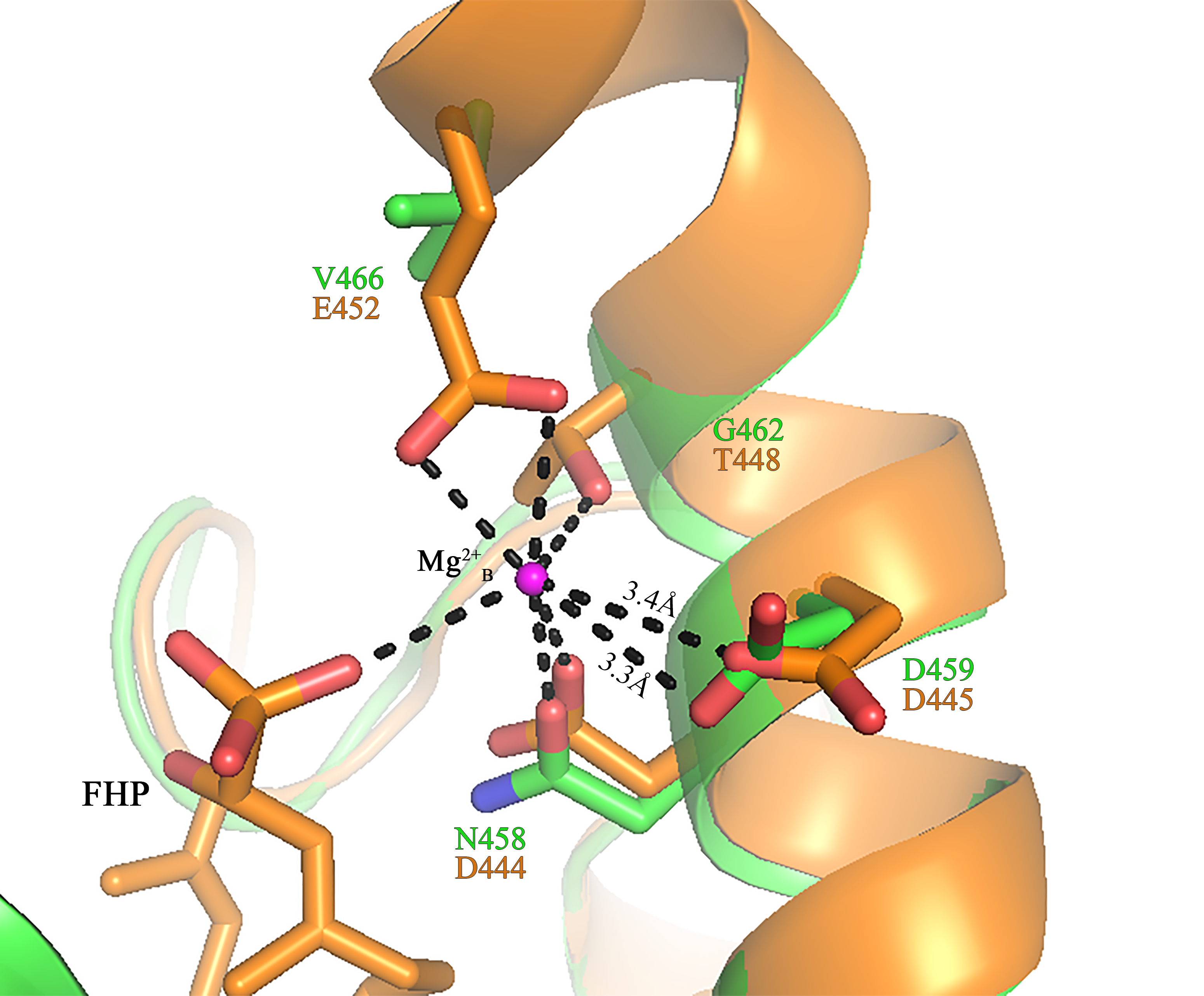

Supplement: Figure S7 — Superimpose of PmSTS (Green) to N. tabacum 5-epi-aristolochene synthase (NtEAS) (PDB:5EAT in brown). The Mg 2+B is shown as magenta sphere. Oxygen and nitrogen atoms are coloured red and blue, respectively. Important distances and likely hydrogen bonds are shown by dashed lines. [file peerj-05-2961-s007.png]

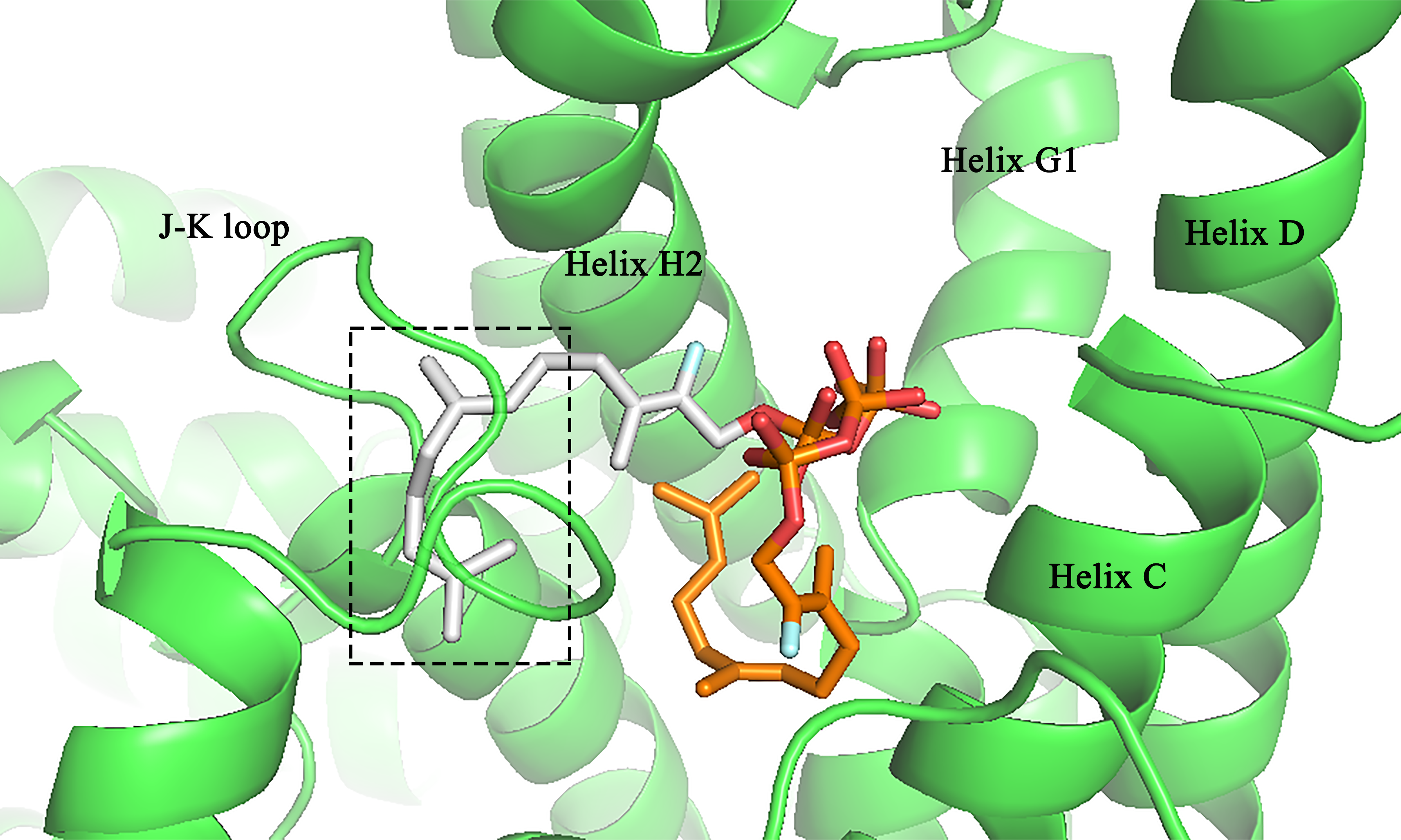

Supplement: Figure S8 — Superimpose of ligand FPF from GaDCS (PDB ID: 3G4F in grey) and ligand FPF from NtEAS (PDB ID: 3M01 in orange). The ligand FPF of GaDCS makes a steric clash with the J-K loop of PmSTS (dashed box). [file peerj-05-2961-s008.png]
