# Supplementary material for: Purification and biochemical characterization of recombinant Persicaria minor β-sesquiphellandrene synthase
Source: PeerJ. 2017 Feb 28;5:e2961. doi: 10.7717/peerj.2961 (PMC5333544; doi:10.7717/peerj.2961)
Supplement: Table S1 [file peerj-05-2961-s009.docx]

| Sesquiterpene | PmSTS | PmSTS_Δ24 |
| --- | --- | --- |
| β-Sesquiphellandrene | 97.4% | 96.2% |
| β-Farnesene | 2.6% | 3.7% |
